# Effect of Intraoperative Regional Anesthesia on Postoperative Outcomes in Pediatric Cardiac Surgery—A Systematic Review of Randomized Controlled Trials

**DOI:** 10.1002/pan.70229

**Published:** 2026-05-25

**Authors:** Katariina Hiisivuori, Heli Salmi, Vesa K. Kontinen, Ilari Kuitunen

**Affiliations:** ^1^ Department of Anesthesia and Intensive Care Helsinki University Hospital Helsinki Finland; ^2^ Pediatric Research Centre University of Helsinki Helsinki Finland; ^3^ Clinicum, Faculty of Medicine University of Helsinki Helsinki Finland; ^4^ Department of Pediatrics Kuopio University Hospital Kuopio Finland; ^5^ Institute of Clinical Medicine University of Eastern Finland Kuopio Finland

**Keywords:** cardiac surgery, pediatric, PICU, postoperative pain, regional anesthesia

## Abstract

**Objective:**

To conduct a meta‐analysis of postoperative outcomes following the use of regional anesthesia in pediatric cardiac surgery.

**Data Sources:**

We searched PubMed (MEDLINE), Web of Science, CINAHL, CENTRAL, and Scopus in December 2024 (PROSPERO: CRD420025635423; registered January 2025).

**Study Selection:**

Two authors screened search results. We included parallel‐grouped randomized controlled trials (RCT) regardless of blinding. Studies comparing adjuvant regional anesthesia (excluding neuraxial anesthesia) with general anesthesia in pediatric patients (0–17 years) having cardiac surgery via midline sternotomy were included.

**Data Extraction:**

Two authors independently extracted the data and assessed risk of bias. The main outcome measures were pain relief using any documented pain score and duration of mechanical ventilation. Other outcome measures sought were postoperative opioid consumption, time to first rescue analgesia, time in pediatric intensive care unit (PICU), length of hospital stay, complications, and patient satisfaction.

**Data Synthesis:**

We identified 15 RCTs with 1055 participants for the analysis. Pain scores were lower during the first 24 h postoperatively, with Modified Objective Pain Scale (MOPS) mean difference (MD) being 0.76 less (MD −0.76; CI −1.19 to −0.32). Duration of mechanical ventilation was 48 min shorter in the regional anesthesia group (MD −48; CI −60 to −36). Postoperative cumulative opioid consumption (morphine equivalents) was 0.21 mg/kg lower (MD −0.21; CI −0.30 to −0.11). No complications related to regional anesthesia were reported.

**Conclusions:**

Our meta‐analysis demonstrates that regional anesthesia in pediatric cardiac surgery reduces postoperative pain, as measured by pain scores and postoperative opioid consumption, leads to shorter mechanical ventilation and PICU stays.

## 1 Introduction

Adequate pain control following cardiothoracic surgery can enhance recovery by reducing metabolic stress and agitation associated with pain, improving hemodynamic stability by enabling earlier restoration of normal respiratory function, and reducing complications by shortening the duration of mechanical ventilation and of overall intensive care. Regional anesthesia offers potential benefits in cardiac surgery, as pediatric patients often have multiple risk factors that may complicate their postoperative course [1].

Neuraxial anesthesia techniques have been shown to reduce pain and stress responses after cardiac surgery. In open‐heart surgery, cardiopulmonary bypass (CPB) requires systemic heparinization, which is why neuraxial anesthesia has remained unpopular. Epidural hematoma is an extremely rare but devastating complication related to neuraxial and paravertebral blocks [2]. Ultrasound‐guided thoracic wall fascia plane blocks and wound infiltration techniques are appealing alternatives to neuraxial anesthesia and are thought to be safer choices with systemic anticoagulation. Each of these techniques has a different risk profile due to approach, depth, and anatomical considerations. Still, they are considered superficial blocks when administered during systemic anticoagulation [3]. Multiple injections and relatively large volumes of local anesthetics also raise concerns about possible infections and local anesthetic toxicity.

We wanted to see whether adjuvant perioperative regional anesthesia, excluding neuraxial anesthesia, improved postoperative pain, recovery, and intensive care outcomes in pediatric cardiac surgery when compared to general anesthesia alone. A previous meta‐analysis found that perioperative regional anesthesia, including neuraxial anesthesia, reduced postoperative pain for up to 24 h [4].

## 2 Methods

We conducted a systematic meta‐analysis on the possible advantages of any form of adjuvant regional anesthesia other than neuraxial anesthesia in children (0–17 years) undergoing cardiac surgery.

In December 2024, we searched the following databases: PubMed (MEDLINE), Web of Science, CINAHL, CENTRAL, and Scopus, using the following search terms: regional anesthesia AND (cardiac or cardiothoracic) AND surgery. We included parallel‐group randomized controlled trials (RCT) regardless of blinding. Additional articles were included if found in the references of included articles and assessed suitable to include for review and analysis. We did not search gray literature, and we excluded non‐English language reports, quasi‐randomized trials, cluster randomized trials, observational studies, and studies that did not report any original data. We excluded gray literature as not being peer‐reviewed and non‐English language studies due to lack of resources for translation of these, while acknowledging the risk that some information might be lost. We did search for major databases, which cover most published randomized trials.

The main outcome measures were pain relief measured by any documented pain score and duration of mechanical ventilation. In addition, the following outcomes were assessed if available:

1. other outcome measures related to pain control: opioid use, cumulative opioid dose, and other pain medication use;
2. other outcome measures related to recovery process: length of hospital stay, length of PICU stay, and duration of sedation;
3. other outcome measures related to complications: mortality, readmission to intensive care, re‐operation, withdrawal syndrome, delirium, and complications related to regional anesthesia administration and;
4. other outcome measures related to patient satisfaction: parental or patient satisfaction and health‐related quality of life.

The main outcome measures found and analyzed were postoperative pain scores and duration of mechanical ventilation. Other outcome measures found and analyzed were opioid consumption, length of hospital stay, length of intensive care stay, and time to first rescue analgesia. Most of the studies used either the Modified Objective Pain Scale (MOPS) or the Face, Legs, Activity, Cry, and Consolability (FLACC) scale for pain assessment.

We used risk ratios (RR) with 95% confidence intervals (CI) for categorized outcomes, and we calculated numbers needed to treat (NNT). For continuous outcomes, we reported mean differences (MD) with 95% CIs.

Two authors (KH and IK) screened abstracts and full texts. The Covidence software was used in the screening and extracting process. Where necessary, agreement was reached by discussion or by reference to a third party. Two authors (IK and KH) performed data extraction independently. The following information was extracted: authors, year of publication, country where the study was conducted, study period, study design, original inclusion criteria, intervention and control, total number of people included in the study, numbers of participants in the patient and control groups, and outcomes.

Version 2 of the Cochrane risk‐of‐bias tool (RoB 2) was used to evaluate the quality of included studies. Risk of bias figures were reported, generated with robvis shinyapp (web‐based application for R applications using robvis R‐package). Two reviewers independently performed risk‐of‐bias assessments. Where necessary, agreement was reached by discussion or by reference to a third party. Publication bias was analyzed by visual examination of funnel plots.

RevMan version 5.4 was used for the meta‐analysis. Data analysis was performed according to the Cochrane Handbook for Systematic Reviews of Interventions guidelines. Depending on the outcomes, either MDs, standardized MDs, mean changes, or standardized mean changes were calculated for continuous outcomes. RRs were calculated for dichotomous outcomes and, if the outcome was rare, we used the Mantel–Haenszel method instead of the DerSimonian and Laird method. Forest plots were presented. We used random‐effects methods in all our analyses due to expected uncontrollable heterogeneity in study populations and study settings. The inconsistency index I^2^ for heterogeneity was calculated and presented alongside the forest plots. We interpreted I^2^ > 40 as high.

The body of evidence was assessed using the Grading of Recommendations Assessment, Development and Evaluation (GRADE) approach, giving ratings of high, moderate, low, or very low. However, in the analyses, we did not downgrade the imprecision estimate based only on dichotomized interpretations of Cis.

Our study protocol, the PRISMA 2020 checklist, and search strategy are available in Supporting Information (Appendices S1–S3).

## 3 Results

**FIGURE 1:**
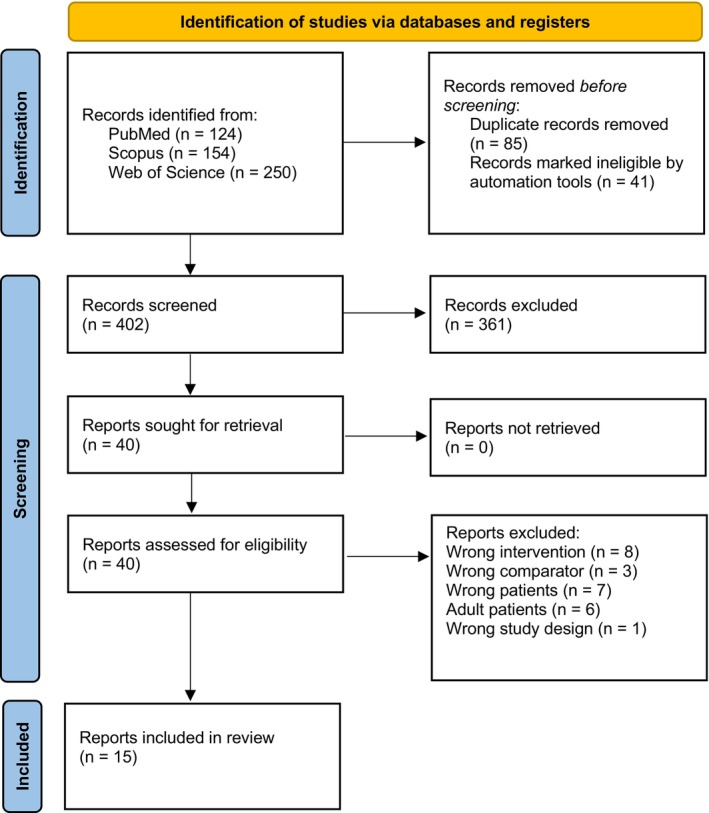
PRISMA flowchart of the study selection process.

We identified 402 reports and, after screening abstracts, 40 were retrieved, of which 25 were excluded on grounds of type of intervention, choice of comparator, type of patients (e.g., adults), or study design. Fifteen studies with 1055 participants were included in the analysis [5, 6, 7, 8, 9, 10, 11, 12, 13, 14, 15, 16, 17, 18, 19] (Figure 1). The characteristics, design, and methods of included studies are summarized in Table S1.

### 3.1 Main Outcome Measures: Pain Reduction and Mechanical Ventilation

**FIGURE 2:**
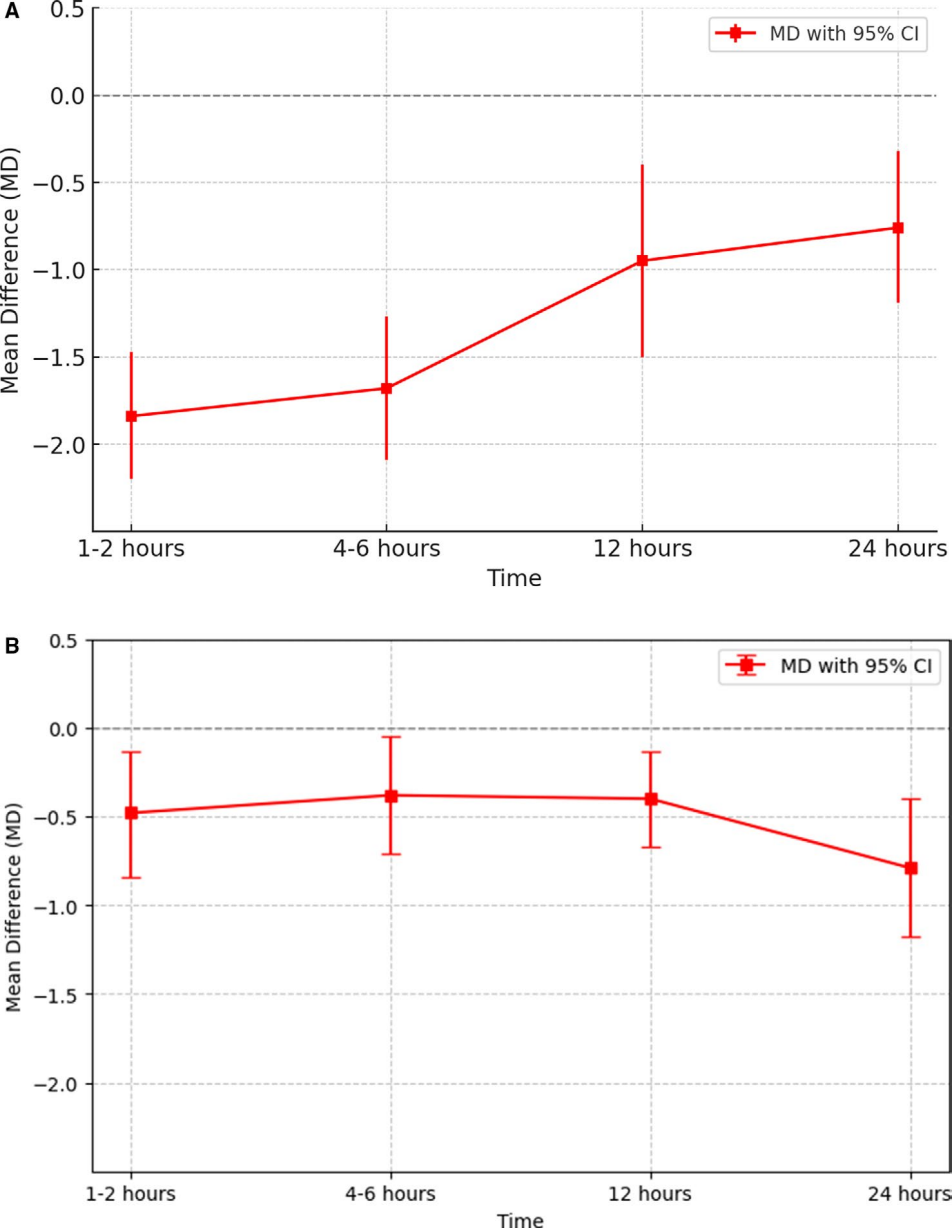
(A, B) Modified Objective Pain Scale (MOPS) and Face, Legs, Activity, Cry, and Consolability Scale (FLACC) for postoperative pain in the first 24 h. Forest plots for all time points available in Supporting Information (Figure S1A,B).

Pain reduction was measured using validated pain scales in thirteen studies with 934 participants. Eleven studies with 827 participants used MOPS as a pain score and two studies with 107 participants used the FLACC scale. One study assessed pain with the COMFORT‐B scale for sedated patients and at three defined time points (after extubation, drain removal, and first mobilization), in addition to using the FLACC scale [14]. Pain scores were significantly lower in the regional anesthesia group during the first 24 h. The MOPS scores were lowest in the first 6 h and significantly lower up to 24 h (Figure 2A). With the FLACC scores, the differences were smaller between groups, still favoring the regional anesthesia group (Figure 2B). Evidence of certainty was graded as high with MOPS scores and moderate with FLACC scores (Table 1).

**TABLE 1:** Summary of findings and evidence certainties assessed according to the GRADE.

Outcome	Number of studies (Number of participants)	Effect estimate	Evidence certainty
Mortality	N/A	N/A	N/A
Pain scores			
MOPS 1–2 h	11 (827)	MD −1.84 (CI −2.20 to −1.47)	High*
FLACC 1–2 h	2 (107)	MD −0.48 (CI −0.84 to −0.13)	Moderate^a^
Mechanical ventilation duration in minutes	13 (934)	MD −48 (CI −60 to −36)	Moderate^b^
Time to first rescue analgesia in hours	8 (632)	MD 3.4 CI (2.4 to 4.4)	High
Postoperative opioid consumption (morphine equivalents) mg/kg			
Total	7 (598)	MD −0.21 (CI −0.30 to −0.11)	Moderate^b^
First 24 h	10 (572)	MD −0.23 (CI −0.32 to −0.14)	Moderate^b^
Length of PICU stay in hours	10 (750)	MD −8.6 (CI −11.3 to −5.9)	Moderate^b^
Length of hospital stay in days	8 (600)	MD −0.64 (CI −1.31 to 0.03)	Moderate^b^

^a^ Downgraded once due to imprecision as only two small studies included.

^b^ Downgraded once due to inconsistency.

* Same evidence certainties to other timepoints measured.

**FIGURE 3:**
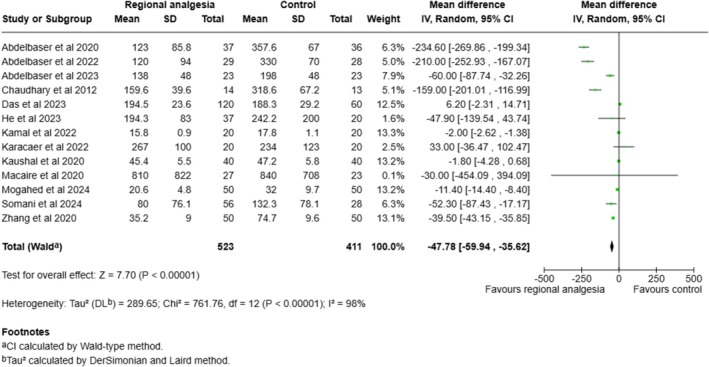
Mechanical ventilation duration in minutes.

Thirteen studies with 934 participants focused on postoperative duration of mechanical ventilation. This was 48 min shorter (MD −47.78; CI −59.94 to −35.62) in the regional anesthesia group (Figure 3). We rated the evidence certainty as moderate (Table 1).

### 3.2 Secondary Outcome Measures and Adverse Effects

**FIGURE 4:**
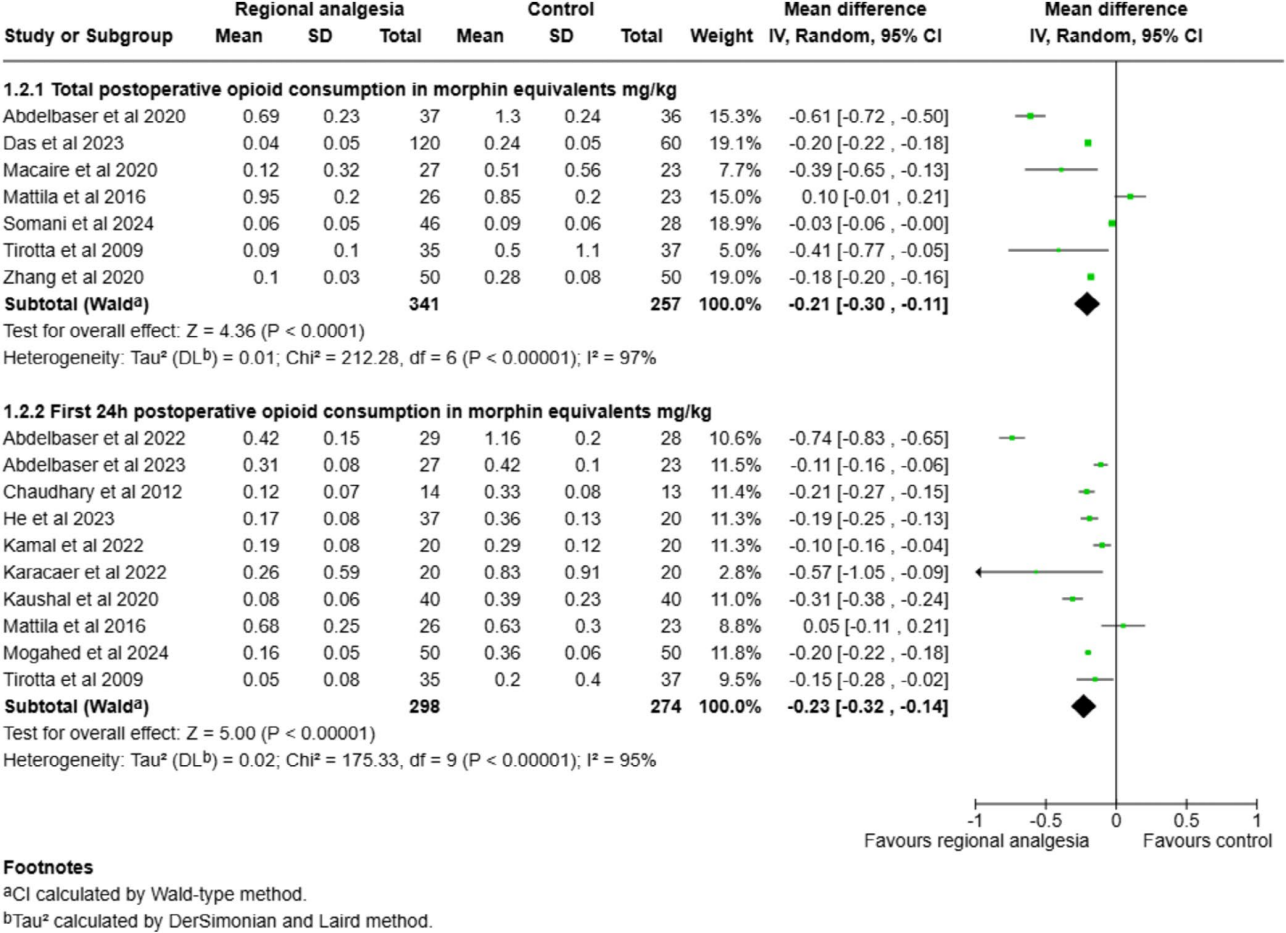
Postoperative cumulative opioid dose in morphine equivalents.

Seven studies with 598 participants measured total postoperative cumulative opioid consumption. We converted opioid doses to morphine equivalents. The regional anesthesia group needed 0.21 mg/kg (MD −0.21; CI −0.30 to −0.11) less morphine equivalents than the control group. Ten studies with 572 participants investigated opioid consumption during the first 24 postoperative hours. In morphine equivalents, opioid consumption was 0.23 mg/kg less (MD −0.23; CI −0.32 to −0.14) in the regional anesthesia group (Figure 4). We rated evidence of certainty moderate (Table 1).

Eight studies with 632 participants measured time to first rescue analgesia after surgery. The regional anesthesia group needed first rescue analgesia more than 3 h later than the control group (MD 3.4; CI 2.4 to 4.4) (Figure S2). Evidence of certainty was rated as high (Table 1).

**FIGURE 5:**
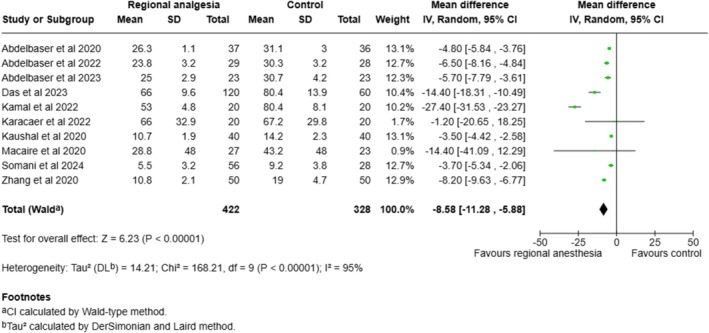
Length of PICU stay in hours.

Length of hospital stay was evaluated in eight studies with 600 participants. There was no significant difference in the length of hospital stay in days (MD −0.68; CI −1.31 to 0.03) (Supplementary Figure 3). Based on ten studies with 750 participants, length of PICU stay was reduced by 8.5 h (MD −8.58; CI −11.28 to −5.88) in the regional anesthesia group (Figure 5). We rated evidence of certainty to both lengths of stay as moderate (Table 1).

Adverse effects related to opioid use (postoperative nausea and vomiting [PONV], pruritus) were reported in thirteen studies with 924 participants. In the study by Macaire et al. [14], PONV was significantly reduced in the regional anesthesia group, but the other studies found no differences between the groups.

Complications related to regional anesthesia techniques were reported in five studies with 293 participants. There were no needle‐related complications (pneumothorax, vascular or neurological injuries, and hematomas) or any complications related to local anesthetic toxicity.

Outcome measures related to complications such as readmission to intensive care, reintubation, withdrawal syndrome, or delirium were rarely reported, and mortality not at all. Kamal et al. [11] studied Pediatric Anesthesia Emergence Delirium (PAED) scores upon admission to the intensive care unit: There was less postoperative agitation in the regional anesthesia group. Outcome measures related to patient satisfaction, parental satisfaction, or quality of life were rarely investigated.

### 3.3 Sensitivity Analyses and Risk‐of‐Bias Assessment

Risk of bias was generally low, and thus, sensitivity analyses excluding high risk‐of‐bias studies did not result in changes to any effect estimates. The risk‐of‐bias assessment is shown in Figure S4.

## 4 Discussion

This systematic review and meta‐analysis of fifteen randomized studies found that the use of regional analgesia in pediatric cardiac surgery reduced pain scores and postoperative opioid consumption and led to shorter mechanical ventilation and duration of PICU stay. Based on study designs, uses of anesthesia, sedation, and nonopioid analgesia were balanced between the intervention and control groups. Local anesthetics were not used to infiltrate wounds or drainage tube insertion sites in control groups.

We found that pain scores were significantly lower during the first postoperative 24 h in the regional anesthesia groups. This was so in nearly all the included studies, and more significant in the early postoperative hours, suggesting a predictable and causal effect of regional anesthesia on pain after pediatric cardiac surgery. The mean difference in pain scores, however, is insufficient to determine whether the effect is clinically relevant. Pain assessment in children, especially nonverbal ones, is challenging, and even when using objective behavioral pain scales, the accuracy and objectivity of pain assessment can be questioned. Objective behavioral scales are also sensitive to level of sedation, developmental stage, and opioid exposure. The best pain measurement tool is self‐reporting, and children over 4 years old are usually able to report their level of pain [20]. Interestingly, none of the studies used self‐reporting tools or utilized parental assessment of pain, both of which would have increased the accuracy of pain assessment.

Poststernotomy pain is known to be moderate to severe, being associated with sternotomy, rib retractions, and insertion of drainage tubes. Many of the included studies concentrated on the management of sternal pain, with management of pain associated with mediastinal and pleural drainage tubes being poorly described. Postoperative pain is usually most severe during the first postoperative day. In most of the included studies, the intervention was a single dose of local anesthetics in the perioperative period, most often timed before surgical incision. Macaire et al. [14] compared intermittent boluses of local anesthetic and saline every 6 h after the initial bolus, up to 48 h postoperatively. Pain scores at 20 and 24 h were lower in the group given intermittent boluses of local anesthetics. Three studies [9, 15, 18] had a bilateral multi‐orifice catheter placed above the periosteum or fascia by the surgeon during sternal closure. All three had a continuous infusion group, and one of the studies also had an intermittent bolus group. Two of the studies found continuous local anesthetic infusion via multi‐orifice catheter effective, while one did not [15]. In the intermittent bolus group, pain scores were lower in the early postoperative period than for continuous infusion [9]. A possible explanation is that fascia plane blocks need a sufficient volume of local anesthetic to work and, with intermittent boluses, sufficient volume may be achieved sooner than with continuous infusion.

Besides placing catheters, it is also possible to prolong the duration of single injection regional anesthesia with adjuvants, such as dexmedetomidine or clonidine, combined with local anesthetic. A meta‐analysis in adults demonstrated that combining dexmedetomidine with local anesthetic in peripheral nerve blocks prolongs duration of analgesia by around 5 h [21]. None of the studies in our meta‐analysis used such adjuvants.

Pain exposure in early life has immediate and long‐term consequences for sensory perception, stress response, and emotional health [22]. Children undergoing cardiac surgery may have had multiple procedures in their early years and have an increased risk of developing prolonged postoperative pain or even chronic postsurgical pain. In adults, chronic pain after sternotomy is common, with a prevalence of 20%–56% [23]. In children, chronic pain after sternotomy has been far less investigated. Lauridsen et al. [24] studied poststernotomy pain in young children and found a prevalence comparable to that reported in adults, with a possible neuropathic component. However, that study did not address the role of regional anesthesia in preventing chronic pain.

The duration of mechanical ventilation was shorter in the regional anesthesia group, although the patient populations consisted of noncomplex cardiac surgery patients where early extubation was in any event expected. Thus, extubation 48 min earlier in the regional anesthesia group may not be clinically significant and may mainly be driven by institutional fast‐track protocols. In the substudies, institutional fast‐track protocols did not differ between groups. The advantages of early extubation include reduced exposure to analgesics and sedation, shorter ICU and hospital lengths of stay, fewer ventilator‐associated complications, and enhanced hemodynamics by increasing systemic venous return and eventually cardiac output [25]. For patients having a total cavopulmonary connection (TCPC), spontaneous ventilation would be especially beneficial [26], but none of the studies included patients having reoperations, so none had a TCPC.

The opioid‐sparing effect of regional anesthesia in the first 24 h was clinically significant. The observed reduction in morphine equivalent use corresponded to a dose that would have been given over 10 h with a moderate infusion rate (20 μg/kg/h). Patients with regional anesthesia also needed the first rescue analgesia later than the control group. Reduced opioid doses probably facilitate earlier extubation by decreasing sedation and respiratory depression. In this meta‐analysis, there was no difference in PONV, except in the study by Macaire et al. [14] where reduced PONV in the regional anesthesia group may be explained by intermittent boluses of local anesthetic up to 48 h.

The study populations were small and, hence, it was not possible to evaluate the real incidence of block‐related complications. No local anesthetic toxicity symptoms were reported, but children are usually anesthetized during the performance of regional anesthesia, so such symptoms could be hidden by general anesthesia. Ultrasound guidance may diminish any local anesthetic systemic toxicity related to regional anesthesia [27].

The strength of this systematic review is its robust methodology, including the fact that all included studies were RCTs with a generally low risk of bias. Twelve of the fifteen studies were from the recent era of ultrasound‐guided regional anesthesia. In the remaining four studies, regional anesthesia was administered by a surgeon during sternal closure. The findings are strengthened by the fact that nearly all results, despite variation in outcome measures, suggested that regional anesthesia provides better outcomes than none.

This systematic review is limited by the small sample sizes, the methodological variations, and the quality of outcome measures in the included studies. As different blocks and several different ways of administering the regional anesthesia were used, it is not possible to identify optimal techniques and patient populations who would benefit the most. True patient‐ or family‐centered outcomes were not used. Initially, we wanted to include mortality as an outcome measure, as it is an outcome of interest in intensive care and postoperative outcomes of pediatric cardiac surgery patients overlap with intensive care outcomes. As none of the studies fulfilling our inclusion criteria used mortality as an outcome, this was not possible. In pediatric cardiac surgery, mortality is largely driven by underlying disease severity and cardiovascular pathology and is unlikely to be affected by the use of regional anesthesia. Also, the underreporting of adverse effects, including system‐related adverse effects such as increased intraoperative resource use, limits the decision of whether or not to use regional anesthesia routinely in pediatric cardiac surgery. Because of the small number of studies, it was not feasible to do any subgroup analysis.

## 5 Conclusions

Adjuvant regional anesthesia seems to be beneficial in pediatric cardiac surgery, but due to heterogeneity and small patient populations in included studies, this should be cautiously interpreted. Adverse effects were reported only infrequently, but it is unlikely that any serious complications would result. There are no data on patient‐centered or long‐term outcomes. Each institution should consider whether the benefits would be worth the resources needed to perform regional anesthesia. Future studies should investigate identifying optimal regional anesthesia techniques and the patient populations which would benefit the most.

## Funding

This work was supported by the Department of Anesthesia and Intensive Care Medicine, Helsinki University Hospital. Lastentautien Tutkimussäätiö.

## Conflicts of Interest

The authors declare no conflicts of interest.

## Supporting information


**Appendix S1:** Study protocol.
**Appendix S2:** PRISMA 2020 checklist.
**Appendix S3:** Search strategy.
**Table S1:** Characteristics, design, and methods of the included studies.
**Figure S1:** (A,B) Pain score forest plots.
**Figure S2:** Time to the first rescue pain medication in hours.
**Figure S3:** Length of hospital stay in days.
**Figure S4:** Risk‐of‐bias assessment.

## Data Availability

The data that support the findings of this study are available in the Supporting Information of this article.
