# Supplementary material for: Effect of Intraoperative Regional Anesthesia on Postoperative Outcomes in Pediatric Cardiac Surgery—A Systematic Review of Randomized Controlled Trials
Source: Paediatr Anaesth. 2026 May 25;36(8):899–907. doi: 10.1002/pan.70229 (PMC13341029; doi:10.1002/pan.70229)

Supplementary file for

**The effect of intraoperative regional anesthesia on postoperative outcomes in pediatric cardiac surgery – a systematic review of randomized controlled trials**

Table of Contents

Appendix 1: Study Protocol 2 - 4

Appendix 2: PRISMA 2020 Checklist 5 - 7

Appendix 3: Search Strategy 8

Supplementary Table 1: Characteristics, design and methods of the included studies 9 -12

Supplementary Figure 1a and b: Pain score forest plots 13-14

Supplementary Figure 2: Time to the first rescue pain medication in hours 15

Supplementary Figure 3: Length of hospital stay in days 16

Supplementary Figure 4: Risk of bias assessment 17

**Appendix 1: Study Protocol**

**The effect of intraoperative regional anaesthesia on postoperative outcomes in paediatric cardiothoracic surgery**

*Ilari Kuitunen, Katariina Hiisivuori, Vesa Kontinen, Heli Salmi*

## Citation

Ilari Kuitunen, Katariina Hiisivuori, Vesa Kontinen, Heli Salmi. The effect of intraoperative regional anaesthesia on postoperative outcomes in paediatric cardiothoracic surgery. PROSPERO 2025 CRD42025635423. Available from <https://www.crd.york.ac.uk/PROSPERO/view/CRD42025635423>.

# REVIEW TITLE AND BASIC DETAILS

## Review title

The effect of intraoperative regional anaesthesia on postoperative outcomes in paediatric cardiothoracic surgery

## Review objectives

Is the use of intraoperative adjuvant regional anaesthesia during paediatric cardiothoracic surgery superior to only general anaesthesia to improve postoperative pain, recovery and intensive care outcomes?

# SEARCHING AND SCREENING

## Searches

Following databases will be used: PubMed (MEDLINE), Web of Science, CINAHL, CENTRAL, and Scopus. The following search term will be used: regional anesthesia AND (cardiac or cardiothoracic) AND surgery. Additional articles will be included, if found in the references of included articles and assessed suitable to include for review and analysis. We will not search grey literature. We will filter non-English reports out.

## Study design

Parallel-grouped randomized controlled trials (RCT) regardless of blinding will be included. Quasi randomized trials and cluster randomized trials will not be included. Observational studies and studies that do not report any original data will be excluded.

# ELIGIBILITY CRITERIA

## Condition or domain being studied

## With the ultrasound-guided regional anaesthesia, regional blocks have become increasingly popular as an additional measures of intra- and postoperative pain management in children undergoing major surgeries. Specifically, the use of regional anaesthesia could have advantages in cardiothoracic surgery, as these children often have multiple risk factors for a complicated postoperative course. Better pain control after cardiac or thoracic surgery could facilitate recovery by decreasing metabolic demands caused by pain and agitation, enable better haemodynamic conditions by restoring normal respiratory function earlier, and decrease complications by shortening the duration of mechanical ventilation and overall intensive care.

## Population

Children (0-17 y) undergoing major cardiothoracic, cardiac or thoracic surgery.

## Intervention(s) or exposure(s)

Any form of regional anaesthesia (excluding neuraxial anesthesia)

## Comparator(s) or control(s)

Other forms of anesthesia (general, neuraxial)

# OUTCOMES TO BE ANALYSED

## Main outcomes

Following outcomes will be assessed as main outcome measures if available: mortality, pain relief by any documented pain score, duration of mechanical ventilation

### *Measures of effect*

Risk ratios with 95% confidence intervals and NNT for categorized outcomes. Mean difference with 95% confidence intervals for continuous outcomes.

## Additional outcomes

In addition, following outcomes will be assessed if information available:
1) other outcome measures related to pain control : opioid use, cumulative opioid dose, other pain medication use
2) other outcome measures related to recovery process: length of hospital stay, length of intensive care unit stay, duration of sedation, costs
3) other outcome measures related to complications: readmission to intensive care, reoperation, withdrawal syndrome, delirium, complications related to regional anaesthesia administration
4) other outcome measures related to patient satisfaction: parental/ patient satisfaction, health related quality of life

### *Measures of effect*

Risk ratios with 95% confidence intervals and NNT for categorized outcomes. Mean difference with 95% confidence intervals for continuous outcomes.

# DATA COLLECTION PROCESS

## Data extraction (selection and coding)

Two authors will screen abstracts and full texts. Covidence software will be used in the screening and extracting process. Third party opinion will be asked in case of disagreement or alternatively agreement between the two screening authors will be sought. Two authors will perform data extraction independently. Following information will be extracted: authors, year of publication, country where the study was conducted, study period, study design, original inclusion criteria, intervention and control, total number of people included in the study, number of patients in the patient group and number of participants in control group, outcomes.

## Risk of bias (quality) assessment

Cochrane risk of bias tool 2.0 will be used to evaluate the quality of included randomized studies. Risk of bias figures will be reported, and these will be generated with robvis shinyapp (web-based application for R applications using robvis R-package). Two reviewers will independently perform the risk of bias assessments. Disagreements will be resolved by third party opinion or mutual agreement.

# PLANNED DATA SYNTHESIS

## Strategy for data synthesis

The RevMan version 5.4 will be used for the meta-analysis. Data analysis will be performed according to Cochrane handbook of systematic reviews guidelines. Depending on the outcomes either mean differences / standardized mean difference or mean change / standardized mean change will be calculated for continuous outcomes. Risk ratios will be calculated for dichotomous outcomes and if the outcome is rare we will use Mantel-Haenszel method, instead of DerSimonian and Laird method. Forest plots will be presented. We will use random-effects methods in all our analysis due to expected uncontrollable heterogeneity in the study populations and study settings. The inconsistency index statistic I² for heterogeneity will be conducted and presented alongside the forest plots. We will interpret I² >40 or more as high.
Publication bias will be analyzed by visual examination of funnel plots and trim-and-fill method.
Sensitivity analyses will be performed with studies judged to have high risk of bias excluded.
The body of evidence will be assessed by using GRADE (Grading of Recommendations, Assessment, Development and Evaluations) and the rating will be high, moderate, low, or very low. However, in the analyses we will not downgrade the imprecision estimate only based on dichotomized interpretation of confidence intervals.

## Analysis of subgroups or subsets

If possible, we will conduct a subgroup analysis based on type of regional anaesthesia, surgery type (cardiac/ thoracic/ cardiothoracic), and whether the surgery has been performed under cardiopulmonary bypass or no

**Appendix 2:** PRISMA 2020 Checklist

| **Section and Topic** | **Item #** | **Checklist item** | **Location where item is reported** |
| --- | --- | --- | --- |
| **TITLE** | | |  |
| Title | 1 | Identify the report as a systematic review. | page 1 |
| **ABSTRACT** | | |  |
| Abstract | 2 | See the PRISMA 2020 for Abstracts checklist. | page 3 |
| **INTRODUCTION** | | |  |
| Rationale | 3 | Describe the rationale for the review in the context of existing knowledge. | page 4 |
| Objectives | 4 | Provide an explicit statement of the objective(s) or question(s) the review addresses. | page 4 |
| **METHODS** | | |  |
| Eligibility criteria | 5 | Specify the inclusion and exclusion criteria for the review and how studies were grouped for the syntheses. | page 5 |
| Information sources | 6 | Specify all databases, registers, websites, organisations, reference lists and other sources searched or consulted to identify studies. Specify the date when each source was last searched or consulted. | page 5 |
| Search strategy | 7 | Present the full search strategies for all databases, registers and websites, including any filters and limits used. | supplementary file appendix 3 |
| Selection process | 8 | Specify the methods used to decide whether a study met the inclusion criteria of the review, including how many reviewers screened each record and each report retrieved, whether they worked independently, and if applicable, details of automation tools used in the process. | page 6 |
| Data collection process | 9 | Specify the methods used to collect data from reports, including how many reviewers collected data from each report, whether they worked independently, any processes for obtaining or confirming data from study investigators, and if applicable, details of automation tools used in the process. | page 6 |
| Data items | 10a | List and define all outcomes for which data were sought. Specify whether all results that were compatible with each outcome domain in each study were sought (e.g. for all measures, time points, analyses), and if not, the methods used to decide which results to collect. | page 7 |
|  | 10b | List and define all other variables for which data were sought (e.g. participant and intervention characteristics, funding sources). Describe any assumptions made about any missing or unclear information. | supplementary table 1 |
| Study risk of bias assessment | 11 | Specify the methods used to assess risk of bias in the included studies, including details of the tool(s) used, how many reviewers assessed each study and whether they worked independently, and if applicable, details of automation tools used in the process. | page 6, supplementary table 2 |
| Effect measures | 12 | Specify for each outcome the effect measure(s) (e.g. risk ratio, mean difference) used in the synthesis or presentation of results. | pages 7-9 |
| Synthesis methods | 13a | Describe the processes used to decide which studies were eligible for each synthesis (e.g. tabulating the study intervention characteristics and comparing against the planned groups for each synthesis (item #5)). | pages 6-7 |
|  | 13b | Describe any methods required to prepare the data for presentation or synthesis, such as handling of missing summary statistics, or data conversions. | pages 6-7 |
|  | 13c | Describe any methods used to tabulate or visually display results of individual studies and syntheses. | pages 6-7 |
|  | 13d | Describe any methods used to synthesize results and provide a rationale for the choice(s). If meta-analysis was performed, describe the model(s), method(s) to identify the presence and extent of statistical heterogeneity, and software package(s) used. | pages 6-7 |
|  | 13e | Describe any methods used to explore possible causes of heterogeneity among study results (e.g. subgroup analysis, meta-regression). | page 13 |
|  | 13f | Describe any sensitivity analyses conducted to assess robustness of the synthesized results. | page 9 |
| Reporting bias assessment | 14 | Describe any methods used to assess risk of bias due to missing results in a synthesis (arising from reporting biases). | page 6, supplementary table 2 |
| Certainty assessment | 15 | Describe any methods used to assess certainty (or confidence) in the body of evidence for an outcome. | page 7 |
| **RESULTS** | | |  |
| Study selection | 16a | Describe the results of the search and selection process, from the number of records identified in the search to the number of studies included in the review, ideally using a flow diagram. | figure 1 |
|  | 16b | Cite studies that might appear to meet the inclusion criteria, but which were excluded, and explain why they were excluded. | - |
| Study characteristics | 17 | Cite each included study and present its characteristics. | page 7, supplementary table 1 |
| Risk of bias in studies | 18 | Present assessments of risk of bias for each included study. | supplementary table 2 |
| Results of individual studies | 19 | For all outcomes, present, for each study: (a) summary statistics for each group (where appropriate) and (b) an effect estimate and its precision (e.g. confidence/credible interval), ideally using structured tables or plots. | pages 7-9. figures 2-4, supplementary figures 1-3 |
| Results of syntheses | 20a | For each synthesis, briefly summarise the characteristics and risk of bias among contributing studies. | pages 7-9 |
|  | 20b | Present results of all statistical syntheses conducted. If meta-analysis was done, present for each the summary estimate and its precision (e.g. confidence/credible interval) and measures of statistical heterogeneity. If comparing groups, describe the direction of the effect. | pages 7-9 |
|  | 20c | Present results of all investigations of possible causes of heterogeneity among study results. | - |
|  | 20d | Present results of all sensitivity analyses conducted to assess the robustness of the synthesized results. | page 9 |
| Reporting biases | 21 | Present assessments of risk of bias due to missing results (arising from reporting biases) for each synthesis assessed. | supplementary table 2 |
| Certainty of evidence | 22 | Present assessments of certainty (or confidence) in the body of evidence for each outcome assessed. | table 1 |
| **DISCUSSION** | | |  |
| Discussion | 23a | Provide a general interpretation of the results in the context of other evidence. | pages 10-13 |
|  | 23b | Discuss any limitations of the evidence included in the review. | pages 12-13 |
|  | 23c | Discuss any limitations of the review processes used. | pages 12-13 |
|  | 23d | Discuss implications of the results for practice, policy, and future research. | page 13 |
| **OTHER INFORMATION** | | |  |
| Registration and protocol | 24a | Provide registration information for the review, including register name and registration number, or state that the review was not registered. | page 3 |
|  | 24b | Indicate where the review protocol can be accessed, or state that a protocol was not prepared. | page 3 |
|  | 24c | Describe and explain any amendments to information provided at registration or in the protocol. | page 5 |
| Support | 25 | Describe sources of financial or non-financial support for the review, and the role of the funders or sponsors in the review. | page 2 |
| Competing interests | 26 | Declare any competing interests of review authors. | page 14 |
| Availability of data, code and other materials | 27 | Report which of the following are publicly available and where they can be found: template data collection forms; data extracted from included studies; data used for all analyses; analytic code; any other materials used in the review. | pages 6-7 |

*From:*  Page MJ, McKenzie JE, Bossuyt PM, Boutron I, Hoffmann TC, Mulrow CD, et al. The PRISMA 2020 statement: an updated guideline for reporting systematic reviews. BMJ 2021;372:n71. doi: 10.1136/bmj.n71. This work is licensed under CC BY 4.0. To view a copy of this license, visit <https://creativecommons.org/licenses/by/4.0/>

**Appendix 3**: Search Strategy

**Search strategy:**

**PubMed:**

regional anesthesia AND (cardiac or cardiothoracic) AND surgery, filter RCT, limit to CHILDREN.

(("regional anaesthesia"[All Fields] OR "anesthesia, conduction"[MeSH Terms] OR ("anesthesia"[All Fields] AND "conduction"[All Fields]) OR "conduction anesthesia"[All Fields] OR ("regional"[All Fields] AND "anesthesia"[All Fields]) OR "regional anesthesia"[All Fields]) AND ("thoracic surgery"[MeSH Terms] OR ("thoracic"[All Fields] AND "surgery"[All Fields]) OR "thoracic surgery"[All Fields] OR ("cardiac"[All Fields] AND "surgery"[All Fields]) OR "cardiac surgery"[All Fields] OR "cardiac surgical procedures"[MeSH Terms] OR ("cardiac"[All Fields] AND "surgical"[All Fields] AND "procedures"[All Fields]) OR "cardiac surgical procedures"[All Fields] OR ("cardiothoracic"[All Fields] AND ("surgery"[MeSH Subheading] OR "surgery"[All Fields] OR "surgical procedures, operative"[MeSH Terms] OR ("surgical"[All Fields] AND "procedures"[All Fields] AND "operative"[All Fields]) OR "operative surgical procedures"[All Fields] OR "general surgery"[MeSH Terms] OR ("general"[All Fields] AND "surgery"[All Fields]) OR "general surgery"[All Fields] OR "surgery s"[All Fields] OR "surgerys"[All Fields] OR "surgeries"[All Fields])))) AND ((randomizedcontrolledtrial[Filter]) AND (allchild[Filter]))

**Translations**

**regional anesthesia:** "regional anaesthesia"[All Fields] OR "anesthesia, conduction"[MeSH Terms] OR ("anesthesia"[All Fields] AND "conduction"[All Fields]) OR "conduction anesthesia"[All Fields] OR ("regional"[All Fields] AND "anesthesia"[All Fields]) OR "regional anesthesia"[All Fields]

**cardiac surgery:** "thoracic surgery"[MeSH Terms] OR ("thoracic"[All Fields] AND "surgery"[All Fields]) OR "thoracic surgery"[All Fields] OR ("cardiac"[All Fields] AND "surgery"[All Fields]) OR "cardiac surgery"[All Fields] OR "cardiac surgical procedures"[MeSH Terms] OR ("cardiac"[All Fields] AND "surgical"[All Fields] AND "procedures"[All Fields]) OR "cardiac surgical procedures"[All Fields]

**surgery:** "surgery"[Subheading] OR "surgery"[All Fields] OR "surgical procedures, operative"[MeSH Terms] OR ("surgical"[All Fields] AND "procedures"[All Fields] AND "operative"[All Fields]) OR "operative surgical procedures"[All Fields] OR "general surgery"[MeSH Terms] OR ("general"[All Fields] AND "surgery"[All Fields]) OR "general surgery"[All Fields] OR "surgery's"[All Fields] OR "surgerys"[All Fields] OR "surgeries"[All Fields]

**SCOPUS**

TITLE-ABS-KEY ( regional AND anesthesia AND ( cardiac OR cardiothoracic ) AND surgery AND randomized ) AND ( LIMIT-TO ( DOCTYPE , "ar" ) ) AND ( LIMIT-TO ( LANGUAGE , "English" ) )

**WEB OF SCIENCE**

regional and anesthesia and (cardiac OR cardiothoracic) and surgery and randomized (All Fields) and Article (Document Types) and English (Languages)

**Supplementary Table 1**: Characteristics, design and the methods of the included studies

|  |  |  |  |  |  |  |  | Patient characteristics at baseline | | | |
| --- | --- | --- | --- | --- | --- | --- | --- | --- | --- | --- | --- |
|  |  |  |  |  |  |  |  | **N of participants** | | **Age** |  |
| **Study** | Operation | Age range | Inclusion criteria | Exclusion criteria | Intervention (I) vs control (C) | Main outcome(s) | Adverse effects reported | Intervention | Control | Intervention | Control |
| **Abdelbaser 2020** | Cardiac surgery (ASD, VSD, CAVC) via median sternotomy | 2-12 y | 2-12 years with L->R intracardiac shunt undergoing elective open-heart surgery via median sternotomy | Refusal  Emergency/redo- surgery,  infection of the skin on the site of puncture, allergy to bupivacaine,  coagulation disorder,  clinically significant liver or kidney disease,  heart failure  moderate to severe pulmonary hypertension | I: US- guided bilateral transversus thoracic muscle plain block with 0.25% bupivacaine 0.4 ml/kg  C: no block | total dose of fentanyl consumption in the first postoperative 24 h after extubation | Yes: pruritus and vomiting similar in studied groups. No patients developed serious block related complications: pneumothorax, local hematoma or sign of local anesthetic  toxicity | 37 | 36 | 7 | 5 |
| **Abdelbaser 2022** | Cardiac surgery (ASD, VSD, CAVC) via median sternotomy | 2-8 y | ASA I-II cardiac surgery via midline sternotomy with CPB for the repair of simple congenital hearts disease | Emergency/Redo-surgery  Preoperative inotropic support  Pulmonary hypertension  Allergy to local anesthetics  Coagulation disorder | I: US-guided bilateral thoracic retrolaminar block with 0.25% bupivacaine 0.4 ml/kg/side  C: Block with 0.9% saline | fentanyl consumption in the first 24 h after extubation | Yes: No block related complications, pruritus and vomiting similar in both groups | 29 | 28 | 4.2 | 3.9 |
| **Abdelbaser 2023** | Cardiac surgery (non- cyanotic congenital heart disease) via median sternotomy | 2-10 y | ASA II surgical repair of simple non-cyanotic congenital heart disease | ASA III-IV,  moderate to severe pulmonary disease,  Redo- surgery  bleeding disorder  thoracic deformity  neurologic deficit  allergy to bupivacaine  local infection at the puncture site | I: US-guided Bilateral mid-point Transverse process to pleura (MTP) block with 0.25% bupivacaine 0.3ml/kg/side  C: No block | Fentanyl consumption in the first postoperative 24 hours | Yes: pruritus and vomiting similar between groups. No block related complications | 23 | 23 | 4.9 | 4.7 |
| **Chaudhary 2012** | Cardiac surgery via median sternotomy | 1-10 y | Cardiac surgery with median sternotomy; all candidates for fast tracking and extubation within 6 hours postoperatively | Emergency/Redo- surgery  EF<35%, LCOS,  preoperative inotropic support, allergy to amide local anesthetics, recurrent ventricular arrhythmias, postoperative complications needing for returning to OR, needing intubation over 10 hours postoperatively | I: Parasternal intercostal block with 0.5% ropivacaine to 2nd-6th intercostal spaces was administered by operating surgeon before sternal closure  C: same volume of saline was administered | Time for extubation; MOPS; the 24hour cumulative fentanyl dosage | No | 14 | 13 | 5.5 | 5.7 |
| **Das 2023** | Cardiac surgery via median sternotomy | 6 mo - 14 y | ASA I or II, elective, candidate for fast tracking | Emergency/Redo-surgery  preoperative inotropic support  LCOS  recurrent ventricular arrhythmias,  allergies to LA,  surgical bleeding that required re-exploration | IA: Pre-sternal multi orifice catheter, 0.375% ropivacaine infusion  IB: Pre-sternal multi orifice catheter, 0.375% ropivacaine bolus  C: did not receive any local anesthetic | Pain (MOPS scale);  Rescue fentanyl consumption | Yes: reported that there were no complications related to catheter insertion | 120  (A 60, B 60) | 60 | A:7.25  B: 7.0 | 7.15 |
| **He 2023** | Median sternotomy | 1 mo  -6 y | Elective median sternotomy | Emergency surgery, infection or anatomical abnormalities at the site of injection, allergic to ropivacaine, previous sternotomies, LCOS, postoperative cardiac function instability or requiring prolonged intubation for more than 12 hours | I1. US-guided bilateral serratus anterior plane block ropivacaine 0.2% 2.5 mg/kg + i.v. opioid (SAPB) performed within 30 min of admission to the cardiothoracic ICU  I2. US-guided bilateral intercostal nerve block 0.2 % ropivacaine 2.5 mg/kg +i.v. opioid (ICNB) performed within 30 min of admission to the cardiothoracic ICU  C: No block | Total opioid requirement up to 24 hours after admission to the ICU | Yes: complications, nausea and vomiting were not observed in both groups | 18 (SAPB),  19 (ICNB) | 20 | 31.9 mo (S), 40.1 mo (I) | 37.6 mo |
| **Kamal 2022** | Cardiac surgery via median sternotomy | 1-5 y | Cardiac surgery via median sternotomy | Past history of LA allergy, previous surgery, coagulopathy, preoperatively critically ill, patients who didn't meet the extubation criteria at the end of the surgery or were reintubated in ICU. | I: US-guided bilateral Pectoral nerve block (PECS II) 0.25 % bupivacaine 0.5 ml/kg after skin closure and wound dressing at the end of the surgery  C: No block | MOPS at 6 hours postoperative | Yes: there was no difference in need for reintubation, respiratory complications or PONV between groups | 20 | 20 | 17.1 mo | 17.05 mo |
| **Karacaer 2022** | Cardiac surgery via median sternotomy | 2-10 y | cardiac surgery via median sternotomy | preop EF <35%, ventricular arrhythmia/dysrhythmia, LCOS, redo or emergency surgery, scoliosis or other anatomic contraindication to ESPB, a history of bleeding diathesis, anticoagulant drug use time of block, known allergy to any drug used during the study | I: US-guided bilateral Erector Spinae Plain Block (ESPB) with 0.25% bupivacaine 2,5 mg/kg  C: No block | Cumulative morphine consumption for the postoperative 24 hours | Yes: no difference in nausea and vomiting between groups | 20 | 20 | 6 | 6 |
| **Kaushal 2020** | Cardiac surgery via midline sternotomy | not specified | ASA I-II non-cyanotic cardiac surgery via midline sternotomy | preop EF<35%, LCOS, recurrent ventricular arrhythmias, preop inotropic support, allergic to amide type LAs, requiring intubation more than 3 hours postoperatively, requiring redo/ emergency surgery | I: US-guided bilateral erector spinae plain block (ESPB) 0.2% ropivacaine 1.5mg/kg/side  C: No block | MOPS at 0,1,2,4,6,8,10 and 12 hours post-extubation | Yes: no difference nausea and vomiting between groups | 40 | 40 | 28.4 mo | 29.83 mo |
| **Macaire 2020** | Cardiac surgery via midline sternotomy | not specified | ASA II elective cardiac surgery via midline sternotomy | refusal, preop EF<35%, ventricular arrhythmia/dysrhythmia, preop inotropic support, redo or emergency surgery, an allergy to amide-type LAs. | I: US-guided bilateral erector spinae plain block (ESPB) T3-T4 (catheter tip to T5) 0.1%/0.2% ropivacaine initial bolus and programmed intermittent bolus (PIB) with 0.1%/0.2 % ropivacaine for 48 hours  C: ESPB initial bolus and PIB with saline | Total opioid consumption at 48 hours after surgery; patients using rescue analgesia with morphine within 48 hours after surgery | Yes: PONV was lower in Programmed Intermittent bolus (PIB) group, no difference in other adverse effects.  Catheter was removed involuntary 15/13% of patients. | 27 | 23 | 23 mo | 27 mo |
| **Mattila 2016** | ASD closure via midline sternotomy | 1-9 y | ASA II-IV patients undergoing ASD repair | history of developmental delay or mental retardation, any other heart defect, known allergy any LAs, clinically significant liver or renal disease | I: Continuous wound infusion with 0.2% ropivacaine postoperatively (catheter was removed 47-54 hours after surgery has ended), catheter inserted by surgeon after sternal closure: tunneled parallel into the sternal wound above periosteum.  C: continuous wound infusion with saline | Morphine consumption during the first 72 hours after surgery | Yes: no difference PONV between groups | 26 | 23 | 4.8 | 5.2 |
| **Mogahed 2024** | Cardiac surgery via midline sternotomy | 4-12 y | 4- 12 y ASA II-III elective cardiac surgery via midline sternotomy | scoliosis, infection at site of LA injection; LVEF <35% with LCOS; recurrent ventricular arrhythmias; preoperative inotropic support, allergy to LAs; history of opioid treatment; patients requiring mechanical ventilation more than 3 hours; re-exploration, redo or emergency surgery, refusal | I: US-guided bilateral Erector spinae plain block at level T6 0.2% ropivacaine 0.4 ml/kg (max 2mg/kg) with adrenaline 2 ug/ml (same syringe)  C: block with saline | Time to the first rescue analgesia | Yes: no difference in PONV, respiratory depression, pruritus or bradycardia. | 50 | 50 | 7.72 | 7.1 |
| **Somani 2024** | Cardiac surgery via midline ssternotomy | 8 mo-10 y | ASA I-II cardiac surgery via midline sternotomy | emergency surgery, redo surgery, preop inotropic support, abnormal coagulation profile, preop EF <35%, LCOS, recurrent ventricular arrhytmias, known allergy to LAs | I1. US-guided bilateral erector spinae plain block (ESPB) at level T3 0.2% ropivacaine 2mg/kg/side  I2. US-guided bilateral multiple injection costo transverse block (MICB) interspaces between T2-T3 and T4-T5 with 0.2% ropivacaine 2mg/kg/side  C: no block | MOPS at 0,1,2,4,6,8,10 and 12 hours post-extubation | No | 1: 28, 2: 28 | 28 | 1: 5.8, 2:  5.9 | 4.3 |
| **Tirotta 2009** | Cardiac surgery via midline sternotomy | 3 mo-16 y | Scheduled cardiac surgigal procedures where ´fast tracking´ was planned (early extubation; preferably in operating room or at least within 6 h on arrival to the CICU. | age < 3 mo, weight < 5kg, allergy to amide local anesthetics, significant liver/renal disease | I: Continuous wound infusion with 0.25 % bupivacaine/levobupivacaine. After sternal closure the catheter was tunneled through chest tubes sites parallel to wound, after skin closure surgeon injected locally LA 0.5 ml/kg including chest tube sites and elastomeric pump with infusion rate 0.5-5 ml/h (0.2-0.4 mg/kg/h). Infusion was terminated and catheter removed after 72 hours or hospital discharge, which ever occurred first.  C: Wound infusion with saline | Postoperative requirements of systemic analgesia, i.v. sedation and pain assessment score (FLACC 3 mo-3 y, Wrong-Baker FACES 3-7 y and NRS over 7 y, for analysis all pain scales normalized range of 0-10) | Yes: Use of antiemetic as a surrogate of PONV. | 35 | 37 | 53.7 mo | 42.1 mo |
| **Zhang 2020** | Cardiac surgery via median sternotomy | 6-60 mo | ASA II-III cardiac surgery via median sternotomy | Allergy to ropivacaine, preop EF < 40%, clinically significant liver/renal disease, redo surgery, emergency surgery, preop inotropic support | I:US-guided transversus thoracis Muscle plain block (TTP) T4-5 interspace 0.2 % ropivacaine 0.75ml/kg  C: block with saline | MOPS at 2,4,8,12,24 and 48 hours after extubation | Yes: no block related complications | 50 | 50 | 25.98 mo | 24.32 mo |

**Supplementary Figure 1A**: Forest plots for MOPS scores


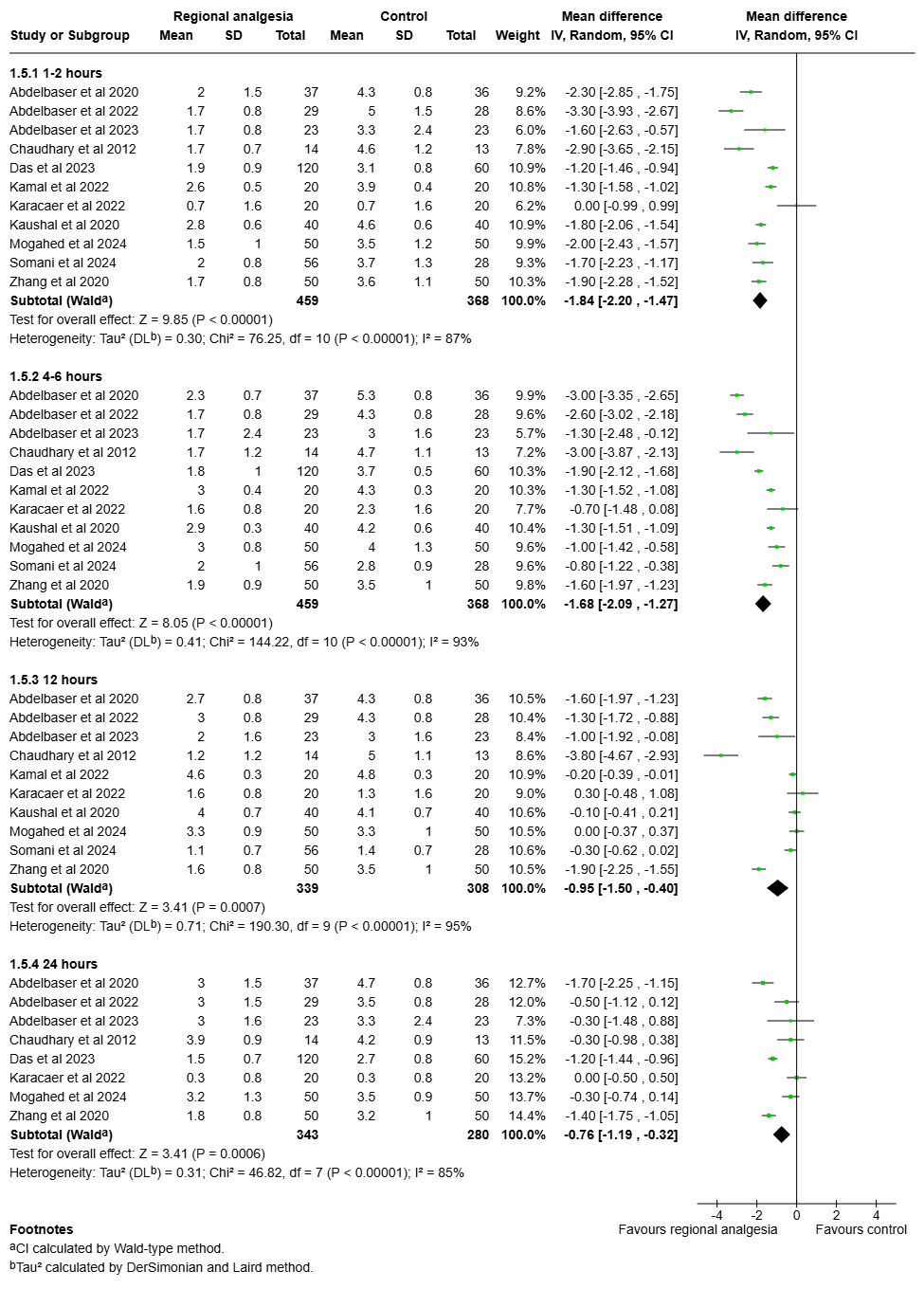


**Supplementary Figure 1B**: Forest plots for FLACC scores


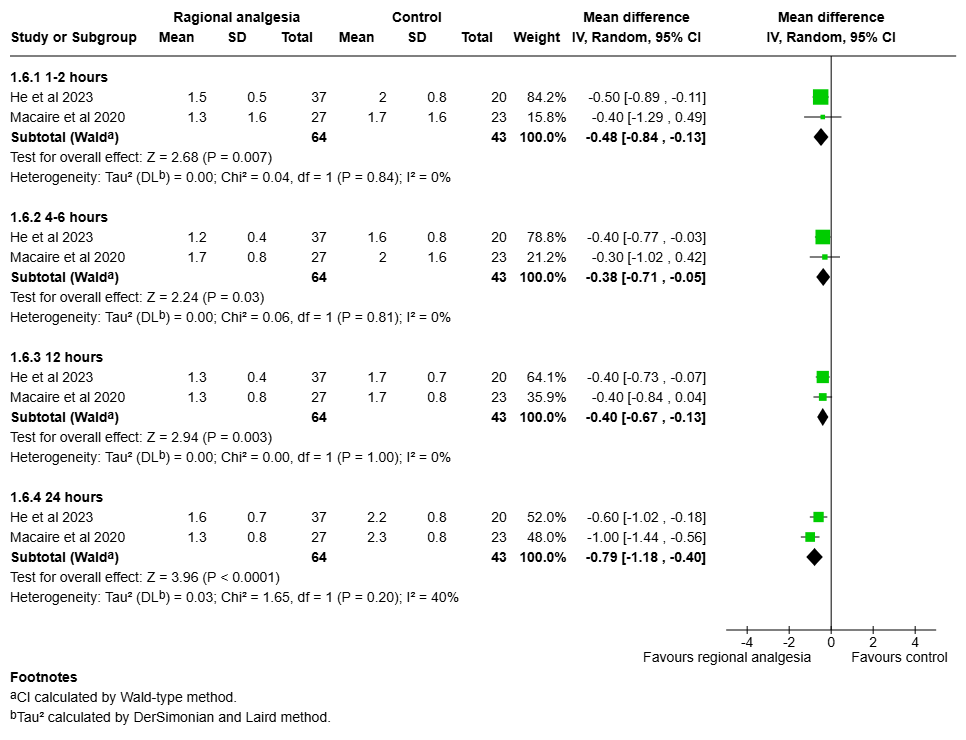


**Supplementary Figure 2**: Time to the first rescue analgesia in hours


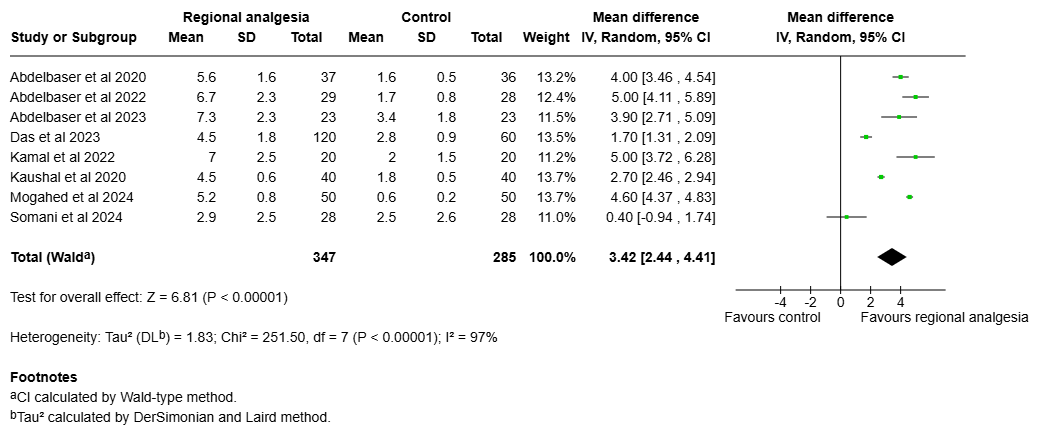


**Supplementary Figure 3**: Length of hospital stay in days


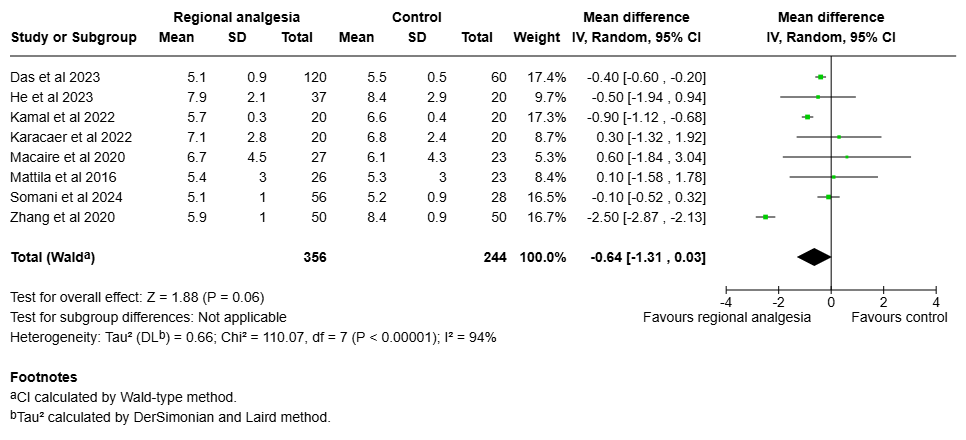


**Supplementary Figure 4**: Risk of Bias Assessment


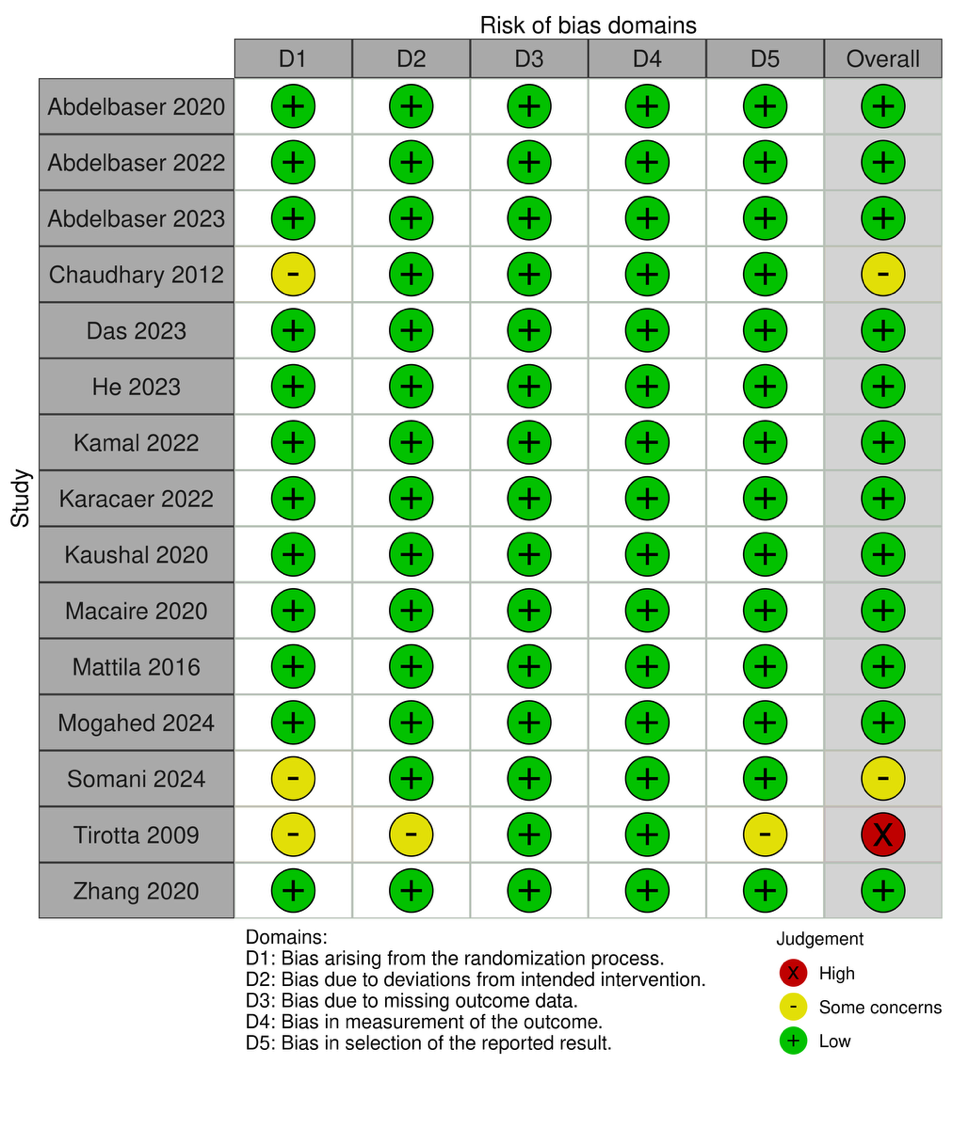

Supplement: Supplementary file 1 — Appendix S1: Study protocol. Appendix S2: PRISMA 2020 checklist. Appendix S3: Search strategy. Table S1: Characteristics, design, and methods of the included studies. Figure S1: (A,B) Pain score forest plots. Figure S2: Time to the first rescue pain medication in hours. Figure S3: Length of hospital stay in days. Figure S4: Risk‐of‐bias assessment. [file PAN-36-899-s001.docx]
